# Crosstalk between the JAK2 and TGF-β1 signaling pathways in scleroderma-related interstitial lung disease targeted by baricitinib

**DOI:** 10.1186/s42358-023-00305-3

**Published:** 2023-05-16

**Authors:** Dandan Wang, Yimei Wei, Lulu Xu, Jie Zhang

**Affiliations:** 1 Division of Respiratory and Critical Care Medicine, Southwest Medical University, Luzhou City, Sichuan Province China; 2 Division of Geriatrics, Chongqing Medical University, Chongqing Municipality, China; 3 Division of Geriatrics, Chongqing General Hospital, Chongqing Municipality, China

**Keywords:** Scleroderma, Interstitial lung disease, JAK2, TGF-β1, Baricitinib

## Abstract

**Background and objective:**

Systemic sclerosis (SSc) is an immune-mediated rheumatic disease characterized by fibrosis and vascular lesions. Interstitial lung disease is an early complication of SSc and the main cause of death from SSc. Although baricitinib shows good efficacy in a variety of connective tissue diseases, its role in systemic sclerosis-related interstitial lung disease (SSc-ILD) is unclear. The objective of our study was to explore the effect and mechanism of baricitinib in SSc-ILD.

**Methods:**

We explored crosstalk between the JAK2 and TGF-β1 pathways. In vivo experiments, SSc-ILD mice model were constructed by subcutaneous injection of PBS or bleomycin (7.5 mg/kg) and intragastric administration of 0.5% CMC-Na or baricitinib (5 mg/kg) once every two days. We used ELISA, qRT‒PCR, western blot and immunofluorescence staining to evaluate the degree of fibrosis. In vitro experiments, we used TGF-β1 and baricitinib to stimulate human fetal lung fibroblasts (HFLs) and assessed protein expression by western blot.

**Results:**

The vivo experiments showed that baricitinib notably alleviated skin and lung fibrosis, decreased the concentration of pro-inflammatory factors and increased those of the anti-inflammatory factors. Baricitinib affected the expression of TGF-β1 and TβRI/II inhibitiing JAK2. In the vitro experiments, following the culture of HFLs with baricitinib or a STAT3 inhibitor for 48 h, the expression levels of TβRI/II decreased. Conversely, with successful inhibition of TGF-β receptors in HFLs, JAK2 protein expression decreased.

**Conclusions:**

Baricitinib attenuated bleomycin-induced skin and lung fibrosis in SSc-ILD mice model by targeting JAK2 and regulating of the crosstalk between the JAK2 and TGF-β1 signaling pathways.

## Introduction

Systemic sclerosis (SSc), also known as scleroderma, is an immune-mediated rheumatic disease that is characterized by fibrosis and vascular lesions on the skin and internal organs [1]. Interstitial lung disease (ILD) is an early complication of SSc [1] and the main cause of death from SSc [2]. The proportion of SSc patients with ILD is as high as 30%. Up to 80% of patients with SSc have pulmonary interstitial abnormalities, and the 10-year mortality rate of systemic sclerosis-related interstitial lung disease (SSc-ILD) patients is as high as 40% [2]. Nintedanib is the first drug approved by the US Food and Drug Administration for the treatment of SSc-ILD [2], but more pharmacological treatments are needed to improve the health of patients with SSc-ILD.

Baricitinib is a JAK1/2 inhibitor that was approved for the treatment of adults with moderate-to-severe rheumatoid arthritis (RA) [3]. Multiple clinical trials and case reports have confirmed that baricitinib can alleviate the clinical symptoms of various diseases, such as psoriasis, atopic dermatitis, and alopecia areata [3–5]. Moreover, studies have found that baricitinib shortens recovery time and accelerates symptom improvement in coronavirus disease 2019 [3–5]. Although baricitinib shows good efficacy in a variety of connective tissue diseases, its efficacy and mechanism in SSc-ILD remain unclear.

## Materials and methods

### Animals and bleomycin administration

Female C57BL/6 mice at 6–8 weeks of age were purchased from the Laboratory Animal Centre of Chongqing Medical University (Chongqing, China). All animal experiments were reviewed and approved by the Ethics Committee of Chengdu Dashuo Biotechnology Co. (Dossy20210119001). The mice were housed in a room with a temperature of 22–26 °C and maintained on a 12 h light–dark cycle with free access to food and water. After adaptive feeding for 1 week, the mice were randomly divided into 3 groups: the control (PBS + 0.5% CMC-Na, *n* = 6), bleomycin (7.5 mg/kg bleomycin + 0.5% CMC-Na, *n* = 13), and baricitinib (7.5 mg/kg bleomycin + 5 mg/kg baricitinib, *n* = 13) groups. The mice were injected subcutaneously with 100 μl of PBS or bleomycin and intragastrically with 100 μl of 0.5% CMC-Na or baricitinib once every two days, and the body weight of the mice was simultaneously recorded. The mice were sacrificed after feeding for 4 weeks, and the dry lung was weighed. Bleomycin was purchased from Nippon Kayaku (Japan), and baricitinib was purchased from AdooQ (China).

### Cell culture

HFLs (ATCC, Shanghai, China) were cultured in F12 medium (FuHeng Biology, Shanghai) supplemented with 10% fetal bovine serum (FBS, Biochannel, Jiangsu, China) and 1% penicillin‒streptomycin. The cells were maintained at 37 °C in a humidified atmosphere containing 5% CO_2_.

### Histological analysis

The left lung and skin were fixed with 4% paraformaldehyde for 24 h and embedded in paraffin. Then, lung and skin sections (4 μm) were prepared and stained with hematoxylin–eosin (HE) or Masson’s trichrome or subjected to immunofluorescence staining. Three randomly selected fields of view of each section were photographed. The HE-stained lung sections were scored for fibrosis severity based on the modified Ashcroft score (0–8 points) [6], and the dermal thickness of the HE-stained skin sections was measured with an Olympus CX23 inverted research microscope. The cumulative collagen content of the Masson’s trichrome-stained lung and skin sections from the mice of all groups was quantified by using ImageJ software. The right lung and skin tissue were stored in a − 80 °C freezer for the other assays.

### ELISA analysis

The mice plasma was tested using ELISA. After centrifugation (3000 rpm, 15 min), we detected the concentrations of IL-1β, IL-6, IL-4 and IL-10 with an ELISA kit following the manufacturer’s protocol (Jianglai Bio, Shanghai).

### Quantitative real-time PCR (qRT‒PCR) analysis

Total RNA was extracted from the lung and skin with TRIzol reagent (Takara, Japan). We obtained cDNA from the total RNA by reverse transcription (Takara), and qRT‒PCR was performed using SYBR Green Master Mix (YEASEN Biotech, China) according to the manufacturer’s protocols. Relative gene expression was quantified relative to expression of the endogenous reference gene (mouse: GAPDH).

### Western blotting

All of the proteins were extracted from lung and skin homogenates or cells by using RIPA (Beyotime, Shanghai) lysis buffer containing phenylmethylsulfonyl fluoride (PMSF, Target Mol, China). The protein concentration was determined using a BCA protein quantification kit (Beyotime, China). After electrophoresis and membrane transfer, the immunoblots were probed with primary antibodies against the following: GAPDH, β-actin, smooth muscle actin (SMA), collagen 4 (Col4), fibronectin (Fn), transforming growth factor beta 1 (TGF-β1), TGF-β receptor type I (TβRI), TGF-β receptor type II (TβRII), janus kinase 2 (JAK2), phospho-JAK2 (p-JAK2), signal tranducer and activator of transcription 3 (STAT3), phospho-STAT3 (p-STAT3), Smad3, and phospho-Smad3 (p-Smad3). The secondary antibodies used were goat anti-rabbit or goat anti-mouse horseradish peroxidase-conjugated antibodies. Enhanced chemiluminescence reagents (4A BIOTECH) were used for detection, and the blots were scanned with CliNX software. Primary antibodies against GAPDH, Col1, Col4, Fn, TGF-β1 and TβRII were purchased from Proteintech (China). Primary antibodies against SMA, JAK2, TβRI, STAT3, p-STAT3, Smad3 and p-Smad3 were purchased from Affinity (China). Primary antibody β-actin was purchased from Huabio (China). Secondary antibodies were purchased from EARTHOX (China).

### Immunofluorescence staining

The paraffin-embedded lung tissues were sectioned, dried and dewaxed. The slices were placed into an antigenic repair solution (Beyotime) and boiled for 20 min. After this, the cells were permeabilized with 0.1% Triton X-100 (Beyotime) for 15 min. The lung sections were blocked with QuickBlock blocking buffer (Beyotime) for 20 min in a humidified chamber. The lung sections were incubated with antibodies against p-JAK2 and TGF-β1 overnight at 4 °C. After washing with 1 × PBS-T 3 times, the lung slices were incubated with FITC-conjugated secondary antibody (EARTHOX). Then, the lung slices were counterstained with DAPI (Beyotime). Fluorescence microscopy was performed by using an Olympus CX23 inverted microscope. The numbers of p-JAK2^+^ and TGF-β1^+^ cells in the lung sections among the groups were statistically analyzed by using ImageJ software.

### RNA interference

For small interfering RNA (siRNA)-mediated knockdown of JAK2 and STAT3 in HFLs, HFLs were transiently transfected with 50 nM siRNA (Ruibo, Guangzhou, China) according to the manufacturer’s protocol. In short, after incubation in F12 medium, which was lacking penicillin and streptomycin, the cells were transfected using LipoGene 2000 Star (Sbjbio, Nanjing) and incubated in Opti-MEM (Gibco, China) for 6 h. The cells were then grown under normal conditions for 72 h.

### Data and statistical analysis

Statistical analysis of all data was performed using GraphPad Prism 8.0.2 software, and data are shown as the means ± SEM. Comparisons were made by one-way analysis of variance (ANOVA) followed by the t test to identify significant differences between groups. Differences for which *p* < 0.05 were considered to be statistically significant.

## Results

### Baricitinib attenuates bleomycin-induced injury and fibrosis

**Fig. 1:**
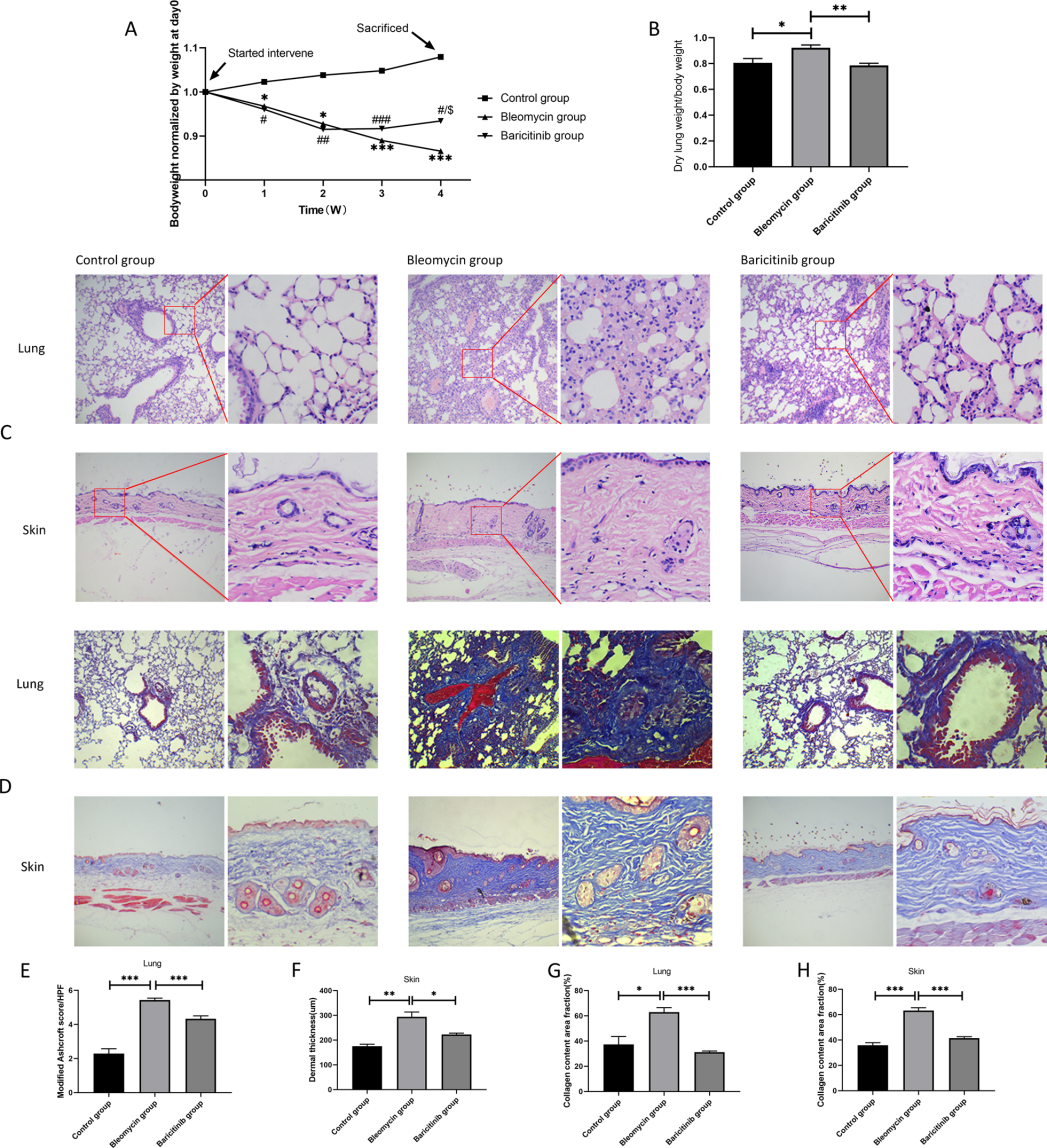
Baricitinib attenuates bleomycin-induced injury and fibrosis. **A** Changes in body weight over time in three groups of mice. *, bleomycin vs control group. #, baricitinib versus control group. $, bleomycin vs baricitinib group (*n* = 6). **B** The levels of lung weight ratios in three groups of mice (*n* = 6). **C** & **D** Lung and skin tissue sections were stained with HE and Masson’s trichrome. The left picture of each group is 100x, and the right enlarged picture of each group is 400x. Statistics of modified ashcroft scores of lung tissue (**E**) and dermal thickness of skin tissue (**F**) among groups in HE staining (*n* = 4). Statistics of lung (**G**) and skin (**H**) collagen content among groups in Masson’s trichrome staining (*n* = 4). Data are presented as the means ± SEM. */#/$ *p* < 0.05, **/## *p* < 0.01, ***/### *p* < 0.001

**Fig. 2:**
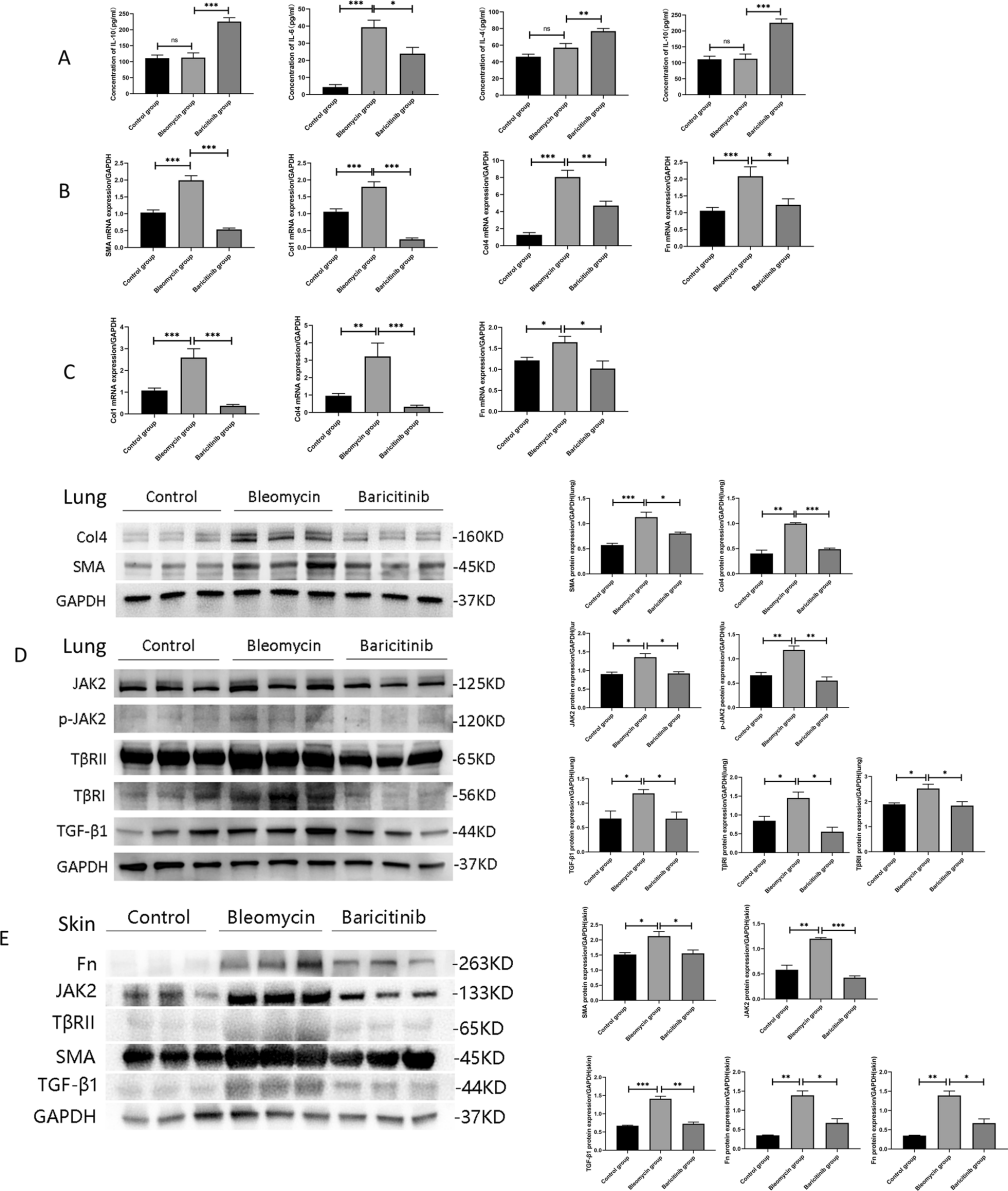
Baricitinib exerted anti-inflammatory and anti-fibrotic effects in SSc-ILD mice through inhibiting both JAK2 and TGF-β1 signalling pathways. **A** The effects of baricitinib on the concentration of inflammatory factors in mice plasma, including IL-1β, IL-6,IL-4 and IL-10 (*n* = 6). mRNA expression of collagen in lung (**B**) and skin (**C**) tissue among three groups (*n* = 6). Western blot analysis of the protein levels of collagen, JAK2, p-JAK2, TGF-β1 and TGF-β receptors in lung (**D**) and skin (**E**) tissue among control, bleomycin and baricitinib groups (*n* = 3). Data are presented as the means ± SEM. * *p* < 0.05, ** *p* < 0.01, *** *p* < 0.001

We first assessed the effects of bleomycin and baricitinib on the body weight of the mice and the lung weight ratio, which was calculated by dividing the dry lung weight by the body weight. The lung weight ratio is an important indicator of pulmonary fibrosis. The higher the lung weight ratio is, the more severe the degree of pulmonary fibrosis is. In our study, we found that bleomycin induced a decrease in body weight and an increase in the lung weight ratio, and baricitinib had the opposite effects (Fig. 1A, B). Persistent inflammatory responses accelerates fibrosis progression. Therefore, we challenged mice with bleomycin and evaluated the anti-inflammatory effects of baricitinib. We also measured the inflammatory factors in mouse plasma by ELISA. Our results showed that bleomycin significantly increased the concentration of the pro-inflammatory factors IL-1β and IL-6 in plasma. Baricitinib significantly decreased the concentration of pro-inflammatory factors and increased the concentration of the anti-inflammatory factors IL-4 and IL-10 (Fig. 2A).

We performed HE and Masson staining of the lung and skin. The results of HE staining showed that bleomycin had significant impacts, causing disruption of the alveolar structure, thickening of the alveolar septa and skin and inflammatory cell infiltration (Fig. 1C), and Masson’s trichrome staining also showed increased collagen deposition (Fig. 1D). However, baricitinib significantly attenuated the above responses. Significant differences in the modified Ashcroft fibrosis score, dermal thickness and collagen content between the three groups were observed (Fig. 1E–H). These results confirmed that baricitinib attenuates bleomycin-induced injury and fibrosis.

### The JAK2 and TGF-β1 signaling pathways are involved in SSc-ILD and were inhibited by baricitinib in the SSc-ILD mice

**Fig. 3:**
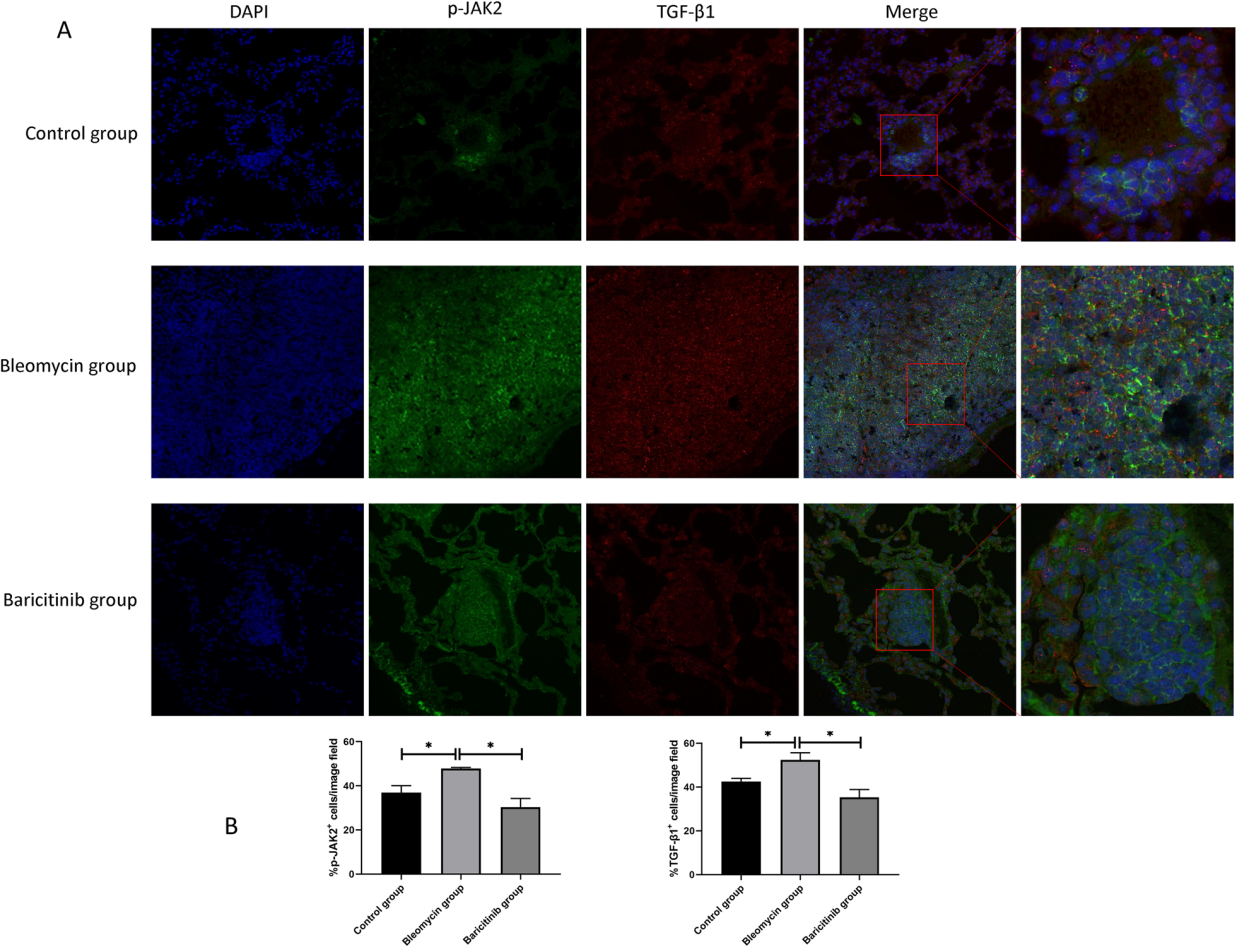
The impacts of baricitinib on JAK2 phosphorylation and TGF-β1 expression in lung tissue in IF assay. **A** Immunofluorescence staining of p-JAK2 and TGF-β1 in the lung tissue among three groups. The left 4 pictures of each group are 200x, and the enlarged picture of each group is 630x. **B** Statistics of p-JAK2^+^ and TGF-β1^+^ cells in lung sections among groups (*n* = 3). Data are presented as the means ± SEM. * *p* < 0.05, ** *p* < 0.01, *** *p* < 0.001

To confirm the effects of baricitinib on fibrosis, we further investigated collagen gene expression and collagen production. The mRNA expression level of collagen in both the skin (Fig. 2C) and lung tissue (Fig. 2B) was significantly downregulated in the baricitinib group relative to the control group of mice with bleomycin-induced SSc-ILD. The collagen content was obviously degraded in the skin (Fig. 2E) and lung (Fig. 2D) in the baricitinib group compared with the bleomycin group. We further investigated whether changes in the collagen content were associated with the JAK2 and TGF-β1 signaling pathways. Our experiments showed that subcutaneous injection of bleomycin significantly increased the protein expression of JAK2, p-JAK2, TGF-β1, and TGF-β receptors in the lung tissue (Fig. 2D) and skin (Fig. 2E), which indicated that the JAK2 and TGF-β1 signaling pathways are extremely likely to be involved in the development and progression of SSc-ILD. The expression of the above proteins was then significantly reduced by treatment with baricitinib. The p-JAK2 and TGF-β1 proteins were examined by immunofluorescence staining (Fig. 3A, B). Bleomycin promoted the activation of JAK2 and increased the expression of TGF-β1, and these effects were then suppressed by baricitinib. These findings again suggested that baricitinib reduces bleomycin-induced pulmonary and skin fibrosis by affecting the JAK2 and TGF-β1 signaling pathways.

### Baricitinib suppresses fibroblast transformation and collagen accumulation by targeting the JAK2 signaling pathway

**Fig. 4:**
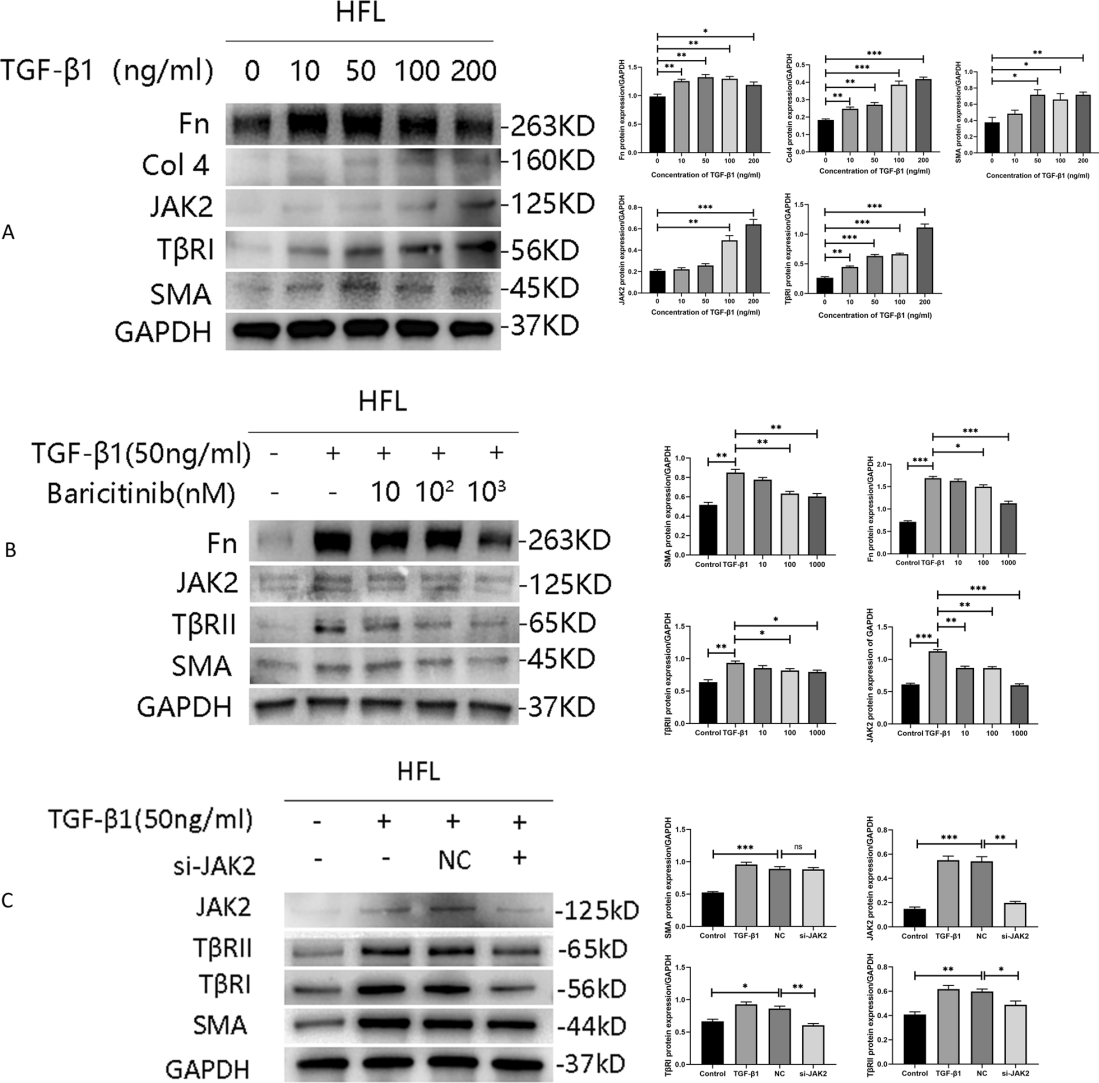
The impacts of TGF-β1, pharmacological inhibition and gene silencing by JAK2 on HFLs. **A** HFLs were treated with different concentrations of TGF-β1 (0, 10, 50, 100 and 200 ng/mL) for 48 h. The protein levels of Fn, Col4, SMA, JAK2 and TGF-β receptors were analyzed by western blot. **B** HFLs were treated with TGF-β1 (50 ng/mL) and baricitinib (10,100 and 1000 nM) for 48 h. The protein levels of Fn, SMA, JAK2 and TGF-β receptors were analyzed by western blot. **C** We used TGF-β1 (50 ng/mL) and 50 nM small RNA interfered JAK2 cultured HFLs. The protein levels of SMA, JAK2 and TGF-β receptors were analyzed by western blot. Data are presented as the means ± SEM. * *p* < 0.05, ** *p* < 0.01, *** *p* < 0.001

We cultured fibroblasts with different concentration of TGF-β1 (0, 10, 50, 100, and 200 ng/ml). After incubation with TGF-β1 for 48 h, the fibroblasts produced more collagen, such as collagen 4, and fibronectin. Furthermore, culture with TGF-β1 promoted the expression of SMA, a myofibroblast marker, which indicated that the fibroblasts had transformed into myofibroblasts (Fig. 4A). We also found that TGF-β1 stimulation increased the expression of JAK2 and TGF-β receptors in a concentration-dependent manner (Fig. 4A), suggesting that the JAK2 and TGF-β1 signaling pathways facilitate fibroblast transformation and collagen deposition. After JAK2 was inhibited, baricitinib reduced SMA expression and collagen deposition in a dose-dependent manner, while TGF-β receptor protein expression decreased significantly (Fig. 4B). We observed similar changes after the JAK2 was silenced (Fig. 4C). These data demonstrated that baricitinib abrogates fibroblast transformation and collagen accumulation.

**Fig. 5:**
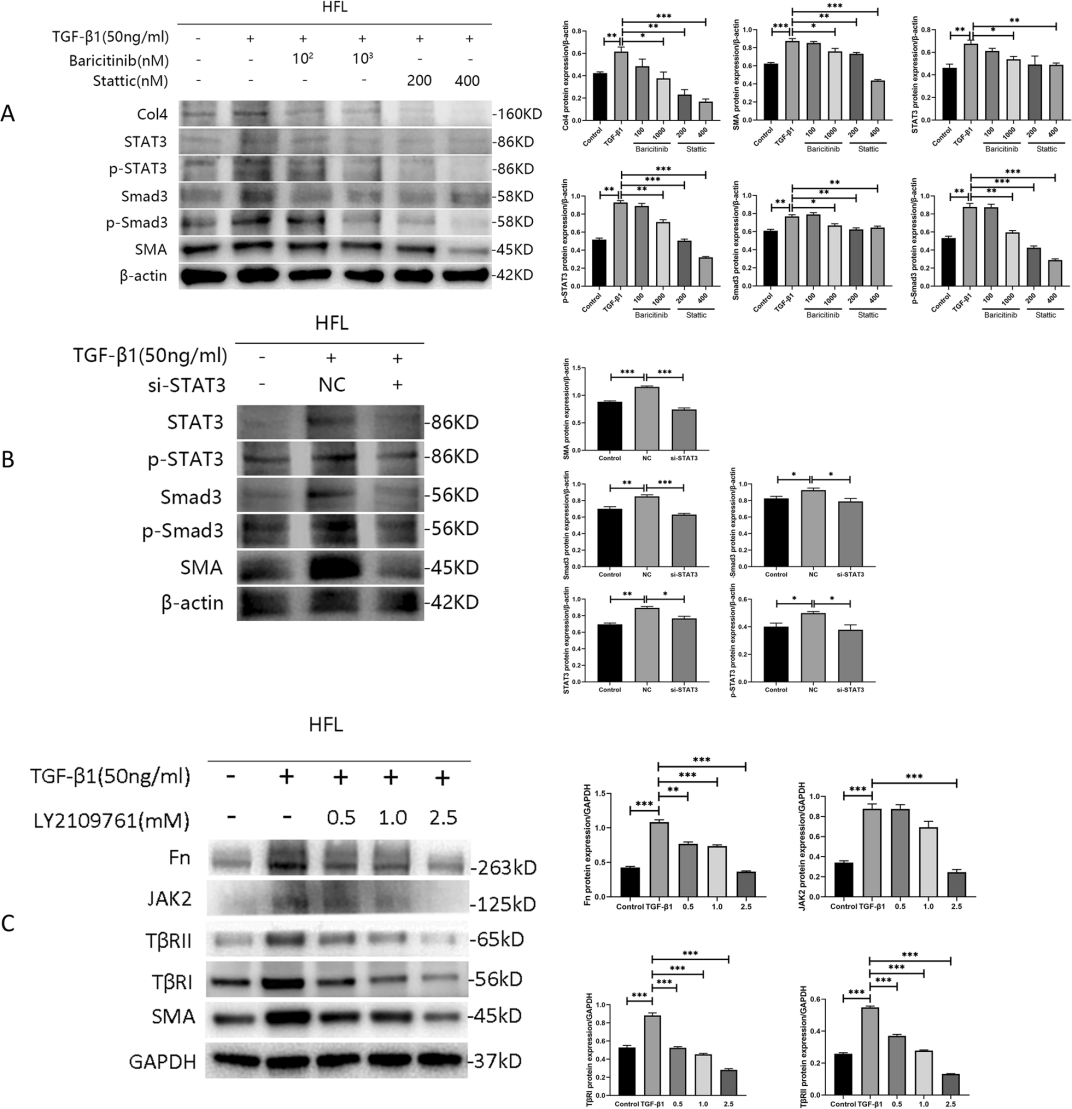
The “crosstalk” between the JAK2 and TGF-β1 signaling pathways in HFLs. **A** HFLs were treated with TGF-β1 (50 ng/mL) and baricitinib (100 and 1000 nM) or stattic (200 and 400 nM) for 48 h. The protein levels of Col4, SMA, STAT3, p-STAT3, Smad3 and p-Smand3 were analyzed by western blot. **B** We used 50 nM small RNA interfered STAT3 cultured HFLs. The protein levels of SMA, STAT3, p-STAT3, Smad3 and p-Smand3 were analyzed by western blot. **C** HFLs were treated with TGF-β1 (50 ng/mL) and LY2109761 (0.5, 1.0 and 2.5 mM) for 48 h. The protein levels of Fn, SMA, JAK2 and TGF-β receptors were analyzed by western blot. Data are presented as the means ± SEM. * *p* < 0.05, ** *p* < 0.01, *** *p* < 0.001

To more specifically explore the mechanism underlying the effects of baricitinib, we detected the downstream proteins of JAK2 and TGF-β1. Our results revealed that the protein expression and activation of collagen, STAT3 and Smad3 were decreased following pharmacological inhibition of JAK2. Stattic is a potent STAT3 inhibitor that inhibits STAT3 phosphorylation. After pharmacological STAT3 inhibition (Fig. 5A) and STAT3 gene silencing (Fig. 5B), Smad3 protein expression and activation also decreased. The above data strongly indicated that baricitinib suppresses fibroblast transformation and collagen accumulation by regulating the JAK2/STAT3 and TGF-β1 signaling pathways.

### Impacts of the TGF-β1 signaling pathway on JAK2

Through pharmacological inhibition and gene silencing, we found that JAK2/STAT3 can regulate the TGF-β1 signaling pathway. However, the effect of the TGF-β1 signaling pathway on JAK2 are uncertain. Therefore, we treated HFLs with LY2109761 to investigate whether the TGF-β1 signaling pathway affects the expression of JAK2. LY2109761 is a dual TβRI/II inhibitor that mainly inhibits TβRI. We found by western blot experiments that LY2109761 successfully inhibited TβRI/II protein to reduce TGF-β1-induced collagen accumulation, inhibited the transformation of fibroblasts into myofibroblasts, and reduced the expression of JAK2 (Fig. 5C). The above results showed that the JAK2/STAT3 and TGF-β1 signaling pathways are regulated by one another.

## Discussion

Due to the high incidence of SSc patients developing ILD, SSc-ILD patients are characterized by high mortality, poor prognosis and few therapeutic drugs. It is therefore of vital important to better understand the pathogenic mechanism of SSc-ILD and to investigate therapeutic agents. The main purpose of this study was to investigate the efficacy and mechanism of baricitinib in mice model of SSc-ILD. With intragastrical administration, baricitinib alleviated the development of skin and lung fibrosis in bleomycin-induced SSc-ILD mice based on the following observations: (i) alleviating dermal and alveolar septal thickening and alveolar structure destruction, (ii) inhibiting TGF-β1 signaling pathways in skin and lung tissue of SSc, (iii) suppressing fibroblast transformation and collagen accumulation in HFLs, (iv) TGF-β receptors influence the expression of JAK2 protein. In summary, the JAK2 and TGF-β1 signaling pathways were found to be involved in the development of SSc-ILD, and its inhibitor baricitinib is a potential therapeutic candidate for the treatment of murine and human SSc.

JAK2 plays an important role in a variety of cytokine signaling pathways and participates in a variety of biological processes [7, 8]. Studies have shown that the levels of p-JAK2 are significantly increased in the lung tissue of patients with IPF [9], and the expression of the JAK2 gene is significantly increased in the lung tissue of patients with RA-usual interstitial pneumonia (RA-UIP) [10]. The association of JAK2 with cardiac fibrosis [11], renal fibrosis [12, 13], IPF [9, 14], and RA-UIP [10] suggests that JAK2 plays an important role in the regulation of tissue fibrosis. In the JAK2/STAT canonical signaling pathway, JAK2 is activated in the cytoplasm when receptor-associated JAK2 is phosphorylated upon ligand binding. Subsequently JAK2 mediates phosphorylation and activation of STAT3, which dimerizes and translocates to the nucleus to regulate gene expression [15]. In addition, the JAK2 signalling pathway also exists as a noncanonical pathway. Recently in myeloproliferative disorders and myeloid malignancies researches, researchers discover JAK2 in the nuclear region of hematopoietic cells, and JAK2 nuclear translocation has been linked to conditions associated with high cell turnover in hematopoietic cells [16]. Additionally, a study about RA- UIP and IPF [10], reveals that JAK2 phosphorylation with two distinct forms of activation: a cytoplasmic form of JAK2 activation in most IPF cases and a nuclear form of p-JAK2 in RA-UIP and a minority of IPF cases. And further assays identified STAT as the downstream transcriptional activator for JAK2-mediated canonical signal transduction and phosphorylation of Tyr41 on histone H3 as the downstream epigenetic regulation site for JAK2-mediated noncanonical signal transduction. In this study, we found that the expression of JAK2 was significantly increased in the skin and lung tissue of bleomycin-induced SSc-ILD mice, which is consistent with the results of previous studies of fibrosis [9, 10, 12] and further confirmed the important role of JAK2 in fibrotic diseases. We found that p-JAK2 is concentrated in the cytoplasm by immunofluorescence experiments. As the result, We speculate that it is very likely that the canonical classical pathway of JAK2 is involved in the pathogenesis and development of SSc-ILD. In vitro studies, our findings indicate that it is highly likely that STAT3 acts as a signalling factor downstream of JAK2 and translocates to the nucleus to regulate the expression of collagen genes.

Increased serum levels of IL-6 in patients with fibrotic disease compared to normal healthy people [17, 18]. In our experiments, we found that IL-6 induced activation of the JAK2 signaling pathway to promote collagen expression and fibroblast-to-myofibroblast transformation in skin and lung tissue. This is in line with previous studies [19, 20]. It is favorable for the treatment of fibrotic diseases to target inhibition of IL-6/JAK2/STAT3 signaling pathway [21, 22]. Surprisingly, the activation of STAT3 induced by IL-6 could further promote the production of cytokines, such as IL-6, TGF-β1 and IL-1β [23]. This creates a vicious cycle that exacerbates fibrosis. Baricitinib, as a JAK2-targeted inhibitor, regulates the secretion of these cytokines, turns a vicious cycle into a virtuous one, prevents further damage to skin and lung tissue, and exerts anti-inflammatory effects.

TGF-β1 is generally recognized as a major regulator of most fibrotic diseases. Additionally, TGF-β1 is essential for normal tissue development and homeostasis of the internal tissue environment [24]. TGF-β1 transmits signals through Smad and non-Smad signaling pathways [25, 26] and other growth factors [5, 27]. These signaling factors promote epithelial-mesenchymal transition and collagen deposition [28]. TGF-β1 stimulates the migration and proliferation of fibroblasts, activates the fibroblast-to-myofibroblast transition and promotes the deposition of extracellular matrix [27, 29]. Activation of the TGF-β receptor triggers fibrous proliferation and promotes the occurrence of fibrotic diseases [25, 27]. In this study, the expression of JAK2, TGF-β1, and TGF-β receptors was increased after treatment with bleomycin and TGF-β1. After baricitinib treatment, the TGF-β receptors and Smad3 protein were reduced, indicating that baricitinib affected TGF-β 1 signaling pathway by inhibiting JAK2 signaling.

In one experiment, inhibiting JAK2 altered the TGF-β1-induced phosphorylation of Smad3 [30], which is important for communication between the TGF-β1 and JAK2 pathways [9]. p-JAK2 has also been reported to be overexpressed in the cytoplasm of skin fibroblasts from patients with SSc, which suggests that JAK2/STAT signaling pathway regulates fibrosis in the skin tissue [31]. In SSc, independent activation of JAK2 and STAT3 by TGF-β1 via SMAD3. Then pharmacologic or genetic inhibition of JAK2 in skin reduces the pro-fibrotic effect of TGF-β1 [31]. Surprisingly, recent evidence suggests that, TGF-β1 could activate STAT3 through a Smad2/3-dependent mechanism to exert profibrotic effects in the fibroblasts of IPF patients, not dependent on JAK2 [32]. In different cells, there extremely likely are physical interactions between STAT3 and TGF-β receptors and between STAT3 and SMAD3 [33]. In our studies, bleomycin-induced SSc-ILD was characterized by an increase of pro-fibrotic mediators IL-1β and IL-6. Therefore inhibition of JAK2 signaling by baricitinib reduced fibrosis and the expression of these mediators. And JAK2/STAT3 signaling pathway is activated in HFLs induced by TGF-β1/Smad3. In conclusion, we speculate that there is a crosstalk between the JAK2 and TGF-β1 signaling pathways, which is involved in SSc-ILD.

Additionally, there is a limitation in our rearch. Although we demonstrated that baricitinib could alleviate skin and lung fibrosis in SSc-ILD mice, this phenomenon was only prophylactic. The efficacy of baricitinib in patients with pre-existing skin and lung tissue fibrosis is not yet available. However, we have found through several experimental results that baricitinib has anti-inflammatory and anti-fibrotic effects, including reducing dermal and alveolar septal thickening, decreasing plasma inflammatory factors and reducing collagen expression in skin and lung tissue. We consequently hypothesize that baricitinib has a positive effect on patients with established SSc-ILD.

Our research confirmed that baricitinib inhibits fibroblast differentiation and collagen accumulation by inhibiting the JAK2/STAT3 signaling pathway, affecting the expression of TGF-β receptors, and regulates the crosstalk between the JAK2/STAT3 and TGF-β1/Smad3 signaling pathways. In addition, baricitinib reduced the degree of skin and lung fibrosis in an SSc-ILD mouse model. This study demonstrates that baricitinib successfully attenuates bleomycin-induced injury and fibrosis of the skin and lung. We conclude that baricitinib can play a role in preventing the occurrence and progression of pulmonary and skin fibrosis in SSc patients. Since baricitinib has been approved for the treatment of RA, which indicates this drug is safe for human [3, 4]. Thus, baricitinib is a promising candidate drug for the prevention of pulmonary complications in patients with SSc.

## Conclusion

Baricitinib attenuated bleomycin-induced SSc-ILD in a mouse model by the targeted inhibition of JAK2 and by regulating crosstalk between the JAK2 and TGF-β1 signaling pathways.

## Data Availability

The raw data required to reproduced the above findings cannot be shared at this time due to the time limitations.
